# Isolation and Identification of F Lavonol Glycosides from *Lathyrus armenus* (Boiss. & Huet)

**DOI:** 10.22037/ijpr.2019.111705.13360

**Published:** 2020

**Authors:** Hajar Heydari, Ozlem Bahadir Acikara, Mehmet Tekin, Gulcin Saltan Iscan

**Affiliations:** a *Ankara University, Faculty of Pharmacy, Department of Pharmacognosy, Ankara, Turkey. *; b *Trakya University, Faculty of Pharmacy, Department of Pharmaceutical Botany, Edirne, Turkey.*

**Keywords:** Endemic, Lathyrus, Secondary metabolites, Flavonoid

## Abstract

In the last five decades study on plant secondary metabolites have been increasing. Higher plants with a wide range of secondary metabolites have been very important in the search of new therapeutic agents. In this study secondary metabolites of *Lathyrus armenus* (Boiss. & Huet) which are endemic in Turkey, were studied. Flavonol glycosides (Rhamnocitrin-3-*O*-rhamninoside, Rhamnetin-3-O-rhamninoside, Rhamnazin- 3-*O*-rhamninoside, kaempferol3-*O*-rhamninoside and, kaempferol-3-*O*-glucosyl (1→2) rhamnoside) were isolated by different chromatographic methods and identified by ^1^H, ^13^C NMR, as well as 2D NMR and Mass spectroscopy techniques from ethyl acetate and aqueous fractions of *L. armenus*’s methanolic extract. This is the first study about secondary metabolites of Turkish *Lathyrus* species.

## Introduction

The family of Leguminosae is one of the largest plant family with 750 genera and more than 18,000 species (1). Leguminosae is divided into three subfamilies namely Papilionoideae, Mimosoideae, and Caesalpinieae. Papilionoideae, and the largest subfamily of Leguminosae which contains 476 genera and 13,860 species is also the most diverse and widely distributed. Includes most of the familiar domesticated food and forage crops and model genetic/genomic species (2). *Lathyrus* (Leguminosae; Papilionoideae) is the largest genus in tribe Fabeae and exhibits an extensive distribution (3). The genus *Lathyrus* L. is divided into three subfamilies and 36 tribes and about 19,325 species of annual and perennial plants (4,5), which are mainly distributed throughout the Northern Hemisphere, like seasonally dry Mediterranean basin and neighboring western Irano-Turanian region. North America and temperate areas of South America are the second regions of diversity and a few species are grown in tropical East Africa. Most members of *Lathyrus* habitant are open woodlands, forest margins, and roadside verges, but littoral, alpine and more drought-tolerant species are also existent (3). In the Flora of Turkey, placed 58 Turkish species (6).

*Lathyrus* species contain various flavonoids, such as quercetin, kaempferol, luteolin, and myricetin. They also contain fatty acids, such as linoleic and linolenic acid and tocopherols (7,8), as well as proanthocyanidins, cyanogenic glucosides (9, 10, 11), phytoecdysteroids (10), pea albumin (12), triterpene saponins (13) and phenolic compounds. 

*L. armenus* is one of endemic species of Turkey. This species is growing in Erzrum, Sivas, Gumushane and Van. In this study the isolation and identification of flavonoits from the whole plants of *L. armenus *(which collected from Sivas) were investigated, and the constituents were isolated and identified based on the spectral data. 

## Experimental

*General*

Methanol, *n*-hexane, CHCl_3_, EtOAC were purchased from Sigma- Aldrich. Silica gel 60 (0.063-0.200 mm), acetonitril and RP-modified Silica Plates were purchased from Merck. The ACE 5, C18 250×4.6 mm, and ACE 10, 250×10 mm HPLC column was used for analysis. Varian Mercury 400MHz Nuclear Magnetic Resonance was used for NMR spectroscopy and Agilent 1100 HPLC series was used for HPLC analysis. 

*Plant material and isolation process*

*L. armenus* was collected from Sivas, Turkey, during the flowering period. Voucher species were identified and the plant sample was deposited for future reference in Ankara University, Faculty of Pharmacy (AEF: 26680). 

The dried and powdered aerial parts of *L. armenus* (800 g) were extracted with methanol by Soxhlet extraction for 12 h. The residue was evaporated under the vacuum and dried (156.185 g) and then partitioned successively between H_2_O and *n*-hexane (47.238 g), CHCl_3_ (1.440 g), EtOAC (5.810 g), the residue or water fraction (97.129 g). 

The EtOAc fraction (5.810 g) was subjected to column chromatography over silica gel with EtOAC:MeOH:H_2_O (100:13.5:10 V:V:V) solvent system to obtain 9 fractions. Sub-fraction 7 was re-chromatographed on reverse phase TLC plates to purify compound **1** (0.133 mg). The MeOH-H_2_O (7:3 V:V) solvent system was used and Rf value for this compound was 0.4. Water fractions (97.129 g) also were fractionated by column chromatography on silica gel using a solvent system composed of EtOAC:MeOH:H_2_O (100:13.5:10 V:V:V) and EtOAC:MeOH (50:50 V:V:V) resulting in 10 sub-fractions. Sub-fraction 6 was purified on a C18 semi preparative HPLC column by Acetonitril: H_2_O mixture to obtain **2** (6 mg), **3** (10 mg), **4** (12 mg), **5** (13.5 mg) compounds. The retention time of these compounds in semi preparative HPLC, were 17,17.3, 19, 21, min respectively. The structure of the isolated compounds has been established by using spectroscopic methods such as ^1^H, ^13^C NMR, as well as 2D NMR and Mass spectroscopy

## Results

In current study, 5 flavonoid derivatives were isolated for the first time from *L.armenus* methanolic extract. 

Compound **1,** yellow amorphous powder, isolated from ethyl acetate fraction, ESI-MS fragmentation displayed [M+H]^+^ m/z 287.14 ion peak, indicating the kaempferol aglycone and [M+H]^+^ m/z 595.32 ion peak for kaempferol glucosyl rhamnoside. The molecular formula was established as C_27_H_30_O_15_ (Kaempferol -3-*O*-glucosyl (1-2) rhamnoside).

Compound **2** yellow amorphous powder, was isolated from water fraction, ESI-MS fragmentation displayed [M+H]^+^m/z 287.14 ion peak, indicating the kaempferol aglycone and [M+H]^+^m/z 741.13 ion peak kaempferol rhamninoside. The molecular formula was established as C_34_H_42_O_20_ (Kaempferol - 3-*O*-rhamninoside).

Compound **3** a yellow amorphous powder, was isolated from water fraction, ESI-MS fragmentation displayed [M+H] ^+^ m/z 317.37 ion peak, indicating the rhamnetin aglycone, and [M+H]^+^m/z 770.71 ion peak rhamnetin rhamninoside. The molecular formula was established as C_34_H_43_O_20_ (Rhamnetin-3-*O*-rhamninoside).

Compound** 4 **a yellow amorphous powder, was isolated from water fraction, ESI-MS fragmentation displayed [M+H]^+ ^m/z 301.24 ion peak, indicating the rhamnocitrin aglycone, and [M+H]^+^m/z 754.35 ion peak rhamnocitrin rhamninoside. The molecular formula was established as C_34_H_43_O_20_ (Rhamnocitrin-3-*O*-rhamninoside). 

**Figure 1:**
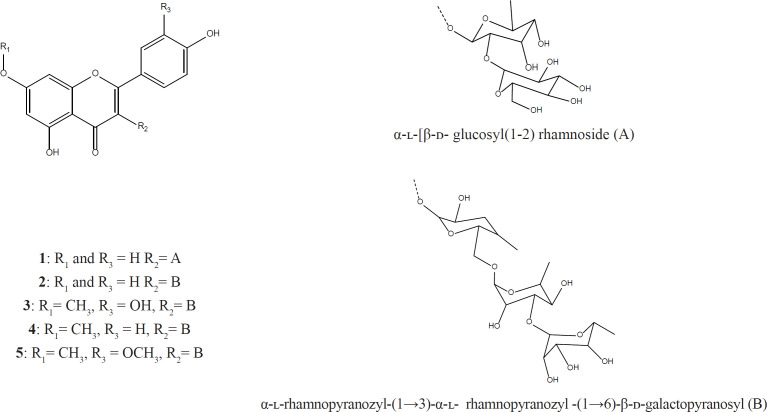
Structure of isolated flavonol glycosides from *L. armenus*

Compound **5** a yellow amorphous powder, was isolated from water fraction, ESI-MS fragmentation displayed [M+H]^+^m/z 331.37 ion peak, indicating the rhamnazin aglycone, and [M+H]^+^m/z 785.40 ion peak rhamnazin rhamninoside. The molecular formula was established as C_34_H_43_O_20_ (Rhamnazin- 3-*O*-rhamninoside). The NMR spectral data measured in methanol-d are shown in Tables 1and 2. The structures of the isolated compounds are shown in Figure 1.

**Table 1:** ^13^C NMR spectral data for isolated compounds (δ in ppm, 400 MHz- CD_3_OD)

	**1**	**2**	**3**	**4**	**5**
-	-	-	-	-	-
C-2	156.87	158.59	159.44	159.73	159.21
C-3	134.38	135.86	136.29	135.99	135.72
C-4	177.55	179.66	179.77	179.74	179.63
C-5	161.15	163.06	162.86	162.74	162.79
C-6	98.69	100.07	99.37	99.26	99.24
C-7	164.35	166.12	167.66	167.46	167.44
C-8	93.66	94.99	93.46	93.35	93.30
C-9	156.39	159.45	158.53	158.46	158.43
C-10	103.90	105.74	106.62	106.53	106.61
C-1'	120.25	122.65	122.85	122.56	122.89
C-2'	130.48	132.54	118.28	116.20	114.72
C-3'	115.30	116.18	145.96	132.61	148.47
C-4'	160.00	161.69	150.35	161.77	151.05
C-5'	115.30	116.18	116.35	116.20	116.06
C-6'	130.48	132.54	123.29	132.61	123.90
7-OCH_3_	-	-	56.74	56.59	56.58
3’- OCH_3_	-	-	-	-	57.03
C-1''	100.78	105.67	106.13	105.53	104.84
C-2''	81.13	73.05	73.36	73.047	73.16
C-3''	69.17	75.12	75.25	75.07	75.05
C-4''	71.56	70.24	70.44	70.24	70.12
C-5''	70.25	75.35	75.46	75.39	75.51
C-6''	17.27	67.53	67.66	67.60	67.53
C-1'''	106.04	101.93	102.10	101.96	102.10
C-2'''	73.75	71.94	72.05	71.93	71.93
C-3'''	76.15	79.62	79.70	79.60	79.60
C-4'''	70.05	73.22	73.36	73.19	73.16
C-5'''	76.54	70.03	70.18	70.03	70.02
C-6'''	60.37	17.99	18.14	17.99	17.99
C-1''''	-	104.02	104.14	104.00	104.14
C-2''''	-	72.17	72.30	72.15	72.15
C-3''''	-	72.23	72.36	72.22	72.22
C-4''''	-	74.11	74.24	74.09	74.06
C-5''''	-	70.03	70.18	70.03	70.02
C-6''''	-	18.02	18.18	18.03	17.99

**Table 2:** ^1^H NMR spectral data for isolated compounds (δ in ppm, 400 MHz).

	**1**	**2**	**3**	**4**	**5**
-	-	-	-	-	-
-	-	-	-	-	-
-	-	-	-	-	-
-	-	-	-	-	-
-	-	-	-	-	-
H-6	6.20 (1H, d, *J*=2.0 Hz)	6.21 (1H, d, *J*=2.0 Hz)	6.33 (1H, d, *J*=2.0 Hz)	6.32 (1H, d, *J*=2.4 Hz)	6.33 (1H, d, *J*=2.4 Hz)
	-	-	-	-	-
H-8	6.37 (1H, d, *J*=2.0 Hz)	6.40 (1H, d, *J*=2.0 Hz)	6.58 (1H, d, *J*=2.0 Hz)	6.56 (1H, d, *J*=2.4 Hz)	6.59 (1H, d, *J*=2.0 Hz)
-	-	-	-	-	-
-	-	-	-	-	-
H-1'	-	-	-	-	-
H-2'	7.76 (2H, d, *J*=8.8 Hz)	8.09 (2H, d, *J*=8.8 Hz)	7.91 (1H, d, *J*=2.0 Hz)	6.88 (1H, d, *J*=8.8 Hz)	8.05 (1H, d, *J*=2.0 Hz)
H-3'	6.94 (2H, d, *J*=8.8 Hz)	6.88 (2H, d, *J*=8.8 Hz)	-	8.11 (1H, dd, *J*=8.8 Hz)	-
H-4'	-	-	-	-	-
H-5'	6.94 (2H, d, *J*=8.8 Hz)	6.88 (2H, d, *J*=8.8 Hz)	6.87 (1H, d, *J*=8.4 Hz)	6.88 (1H, d, *J*=8.8 Hz)	6.90 (1H, d, *J*=8.8 Hz)
H-6'	7.76 (2H, d, *J*=8.8 Hz)	8.09 (2H, d, *J*=8.8 Hz)	7.62 (1H, dd, *J*=8.0, 2.0 Hz)	8.11 (1H, dd, *J*=8.8 Hz)	7.63 (1H, dd, *J*=8.4, 2.0 Hz)
7-OCH_3_	-	-	3.88, s	3.87, s	3.90,s
3’- OCH_3_	-	-	-	-	3.96, s
H-1''	5.71 (1H, d, *J*=1.6 Hz)	5.03 (1H, d, *J*=7.6 Hz)	5.09 (1H, d, *J*=8.0 Hz)	5.06 (1H, d, *J*=8.0 Hz)	5.26 (1H, d, *J*=7.6 Hz)
H-2''	3.20-4.28	3.75 (1H, m)	3.41, 3.83 (1H, m)	3.79 (1H, m)	3.41- 3.92
H-3''		3.54 (1H, m)	3.58 (1H, m)	3.54 (1H, m)	
H-4''		3.81 (1H, m)	3.81 (1H, m)	3.81 (1H, m)	
H-5''		3.64 (1H, brs)	3.68 (1H, m)	3.69 (1H, m)	
H-6''	0.93 (3H, d, *J*= 6.4 Hz)	3.42, 3.74 (1H, m)	3.42; 3.74 (1H, m)	3.41, 3.72 (1H, m)	
H-1'''	4.41 (1H, d, *J*=7.6 Hz)	4.50 (1H, d, *J*=1.2 Hz)	4.50 (1H, d, *J*=1.6 Hz)	4.50 (1H, d, *J*=1.6 Hz)	4.50 (1H, d, *J*=1.2 Hz)
H-2'''	3.20-4.28	3.68 (1H, m)	3.66 (1H, m)	3.68 (1H, m)	3.41- 3.92
H-3'''		3.59 (1H, m)	3.58 (1H, m)	3.58 (1H, m)	
H-4'''		3.4 (1H, m)	3.41, 3.83 (1H, m)	3.41 (1H, m)	
H-5'''		3.69, 3.54 (1H, m)	3.55, 3.66 (1H, m)	3.54, 3.69 (1H, m)	
H-6'''		1.18 (3H, d, *J*= 5.6 Hz)	1.18 (3H, d, *J*= 6.4 Hz)	1.17 (3H, d, *J*= 6.0 Hz)	1.16 (3H, d, *J*= 6.0 Hz)
H-1''''		4.92 (1H, d, *J*=2.0 Hz)	4.91 (1H, d, *J*=1.6 Hz)	4.89 (1H, brs)	4.9 (s, 1H)
H-2''''	-	3.92 (1H, m)	3.92 (1H, m)	3.92 (1H, m)	3.41- 3.92
H-3''''	-	3.71 (1H, m)	3.72 (1H, m)	3.72 (1H, m)	
H-4''''	-	3.32 (1H, m)	3.32 (1H, m)	3.37 (1H, m)	
H-5''''	-	3.69, 3.54 (1H, m)	3.69, 3.56 (1H, m)	3.54, 3.69 (1H, m)	
H-6''''	-	1.14 (3H, d, *J*= 6.0 Hz)	1.12 (3H, d, *J*= 6.4 Hz)	1.12 (3H, d, *J*= 6.4 Hz)	1.10 (3H, d, *J*= 6.4 Hz)

## Discussion

The *Lathyrus* genus plants have endemic importance due to their usage as a food, fodder and ornamental crops. This species of plants contains approximately 25% of protein, and they are similar to other commonly used grain legumes, such as peas and fava beans. However, nonprotein amino acid (β-N-oxalyl-l-α, β-diaminopropionic acid ( β-ODAP)) also is found in *Lathyrus* species in low concentration. Literature data have demonstrated that *Lathyrus* species contain functional compounds including phenolics with antioxidant activity. 

Related to chemistry of *Lathyrus* species it has been reported that quercetin, kaempferol, and their glycosides such as 3-*O*- glucosides, 3-rutinoside, 3-3-glucoside, 3,7- glucoside, 3-galactoside, 3-sophoroside, 7- glucoside, 3-robinobioside and 3-lathyroside-7-rhamnoside were isolated (8).

Further studies on *L. cicera *released that seeds contain 37 glycosylated flavonoids which were identified by HPLC-DAD-ESI/MS method. Kaempferol glycosides were detected as the main glycosides and quercetin, isorhamnetin, apigenin as well as luteolin glycosides were also determined (14).

*L. digitatus* aerial parts content of phenolic acids and flavonoids were determined. Most of the flavonoids were identified as kaempferol and quercetin derivatives according to their mass fragmentations (15). 

Flavonoids are omnipresent in higher plants, and improve plant–microbe interactions, plant–animal interactions. Flavonoids prevalence in plants make them important components in the control of inflammation and cancer prevention (16). Flavonoids are the most widely distributed secondary natural metabolites ,found in the plants occuring in free forms or as glycosides with polyphenolic structure. Becouse of the biochemical and antioxidant properties of flavonoids, these classes of secondary metabolites are associated with wide rang of health-promoting effects such as NF-_K_B activation, aldose reductase inhibition, insulin receptor activator, anti-carcinogenic, anti-mutagenic , etc. There are so many *in-vivo* and *in-vitro* studies about usefulness of flavonoids. They are using as nutraceutical, pharmaceutical, medicinal, and cosmetical pupose. (17, 18, 19). Kaempferol and quercetin and their glycosides were present in 36 of 38 *Lathyrus* species. According to the litrature survey Flavonoids with 3-sophoroside-7-glucoside, 3-robinobioside 3-sophoroside and the 3-lathyroside-7-rhamnoside moiety were isolated from this genus. of Cicer, Lens, Pisum and Vicia indicate that each genus has a distinctive flavonoid profile (8).

The present paper describes NMR study and structure of Rhamnocitrin-3-*O*-rhamninoside, Rhamnetin-3-*O*-rhamninoside, Rhamnazin- 3-*O*-rhamninoside, Rhamnazin- 3-*O*-rhamninoside and Kaempferol-3-*O*-glucosyl (1-2) rhamnoside isolated from *L. armenus*. Kaempferol-3-*O*-glucosyl (1-2) rhamnoside was isolated from ethyl acetate fraction and the other compound was isolated from aqueous fraction of methanolic extract of *L. armenus *(Figure 1). 

^1^H, ^13^C- NMR, HMBC, HSQC, COSY, and TOCSY data of glycosides moiety is clearly indicated the presence of rhamninoside in **2**,**3**,**4**,**5** compounds and glucosyl (1-2) rhamnoside in compound **1**. The obtained data was compared with literature and approved. **2**,**3**,**4**,**5** compounds were isolated from *Rhamnus* species (20, 21) and compound **1 **was isolated from *Ginkgo biloba *and* Hymenophyllum crispatum *(22,23).

## Conclusion

*Lathyrus* species are used as food and nutrient supplements in traditional diets all over the world. These plants have high content of polyphenol and antioxidant compounds. In this study *L. armenus* was selected to screen for its flavonoid contents. Flavonoids with rhamninose and rhamnoside moiety were detected for the first time in *Lathyrus* genus. The best of our knowledge is searching for bioactive compounds from endemic species which is very important, climate and geografic conditions force plants to produce different secondary metabolites. Furture, studies should be performed to reseal *Lathyrus* genus chemistry in detail. 
